# Comment on: Low Health Literacy Exists in the Inflammatory Bowel Disease Population and Is Disproportionately Prevalent in Older African Americans

**DOI:** 10.1093/crocol/otaa079

**Published:** 2020-10-12

**Authors:** Lauren S Languido, Jessica K Salwen-Deremer

**Affiliations:** 1 Department of Psychiatry, Dartmouth-Hitchcock Medical Center, Lebanon, New Hampshire, USA; 2 Department of Medicine, Section of Gastroenterology, Dartmouth-Hitchcock Medical Center, Lebanon, New Hampshire, USA

There is growing interest in the role of health literacy in chronic disease management and prevention, especially because it has been identified as a modifiable predictor of disease course and severity.^1^ Health literacy is frequently defined as one’s capacity to read, process, and comprehend health information, to carry out medical instructions, and to make decisions about one’s health.^2^ Low health literacy (LHL) in particular has been associated with adverse health outcomes among patients living with a chronic illness (eg, HIV, diabetes mellitus, and cancer), including increased hospitalization and readmission rates, poorer quality of life, difficulty adhering to medical recommendations (eg, reading and interpreting labels and instructions), and higher mortality rates.^3, 4^ Among patients with inflammatory bowel diseases (IBD), emerging research demonstrates that LHL rates are fairly high, and that they are associated with poor overall health outcomes, such as increased symptom burden in Crohn’s disease and depressive symptoms in general.^5, 6^

LHL is also common in racial/ethnic minorities (eg, Latinx, African Americans, and Asians),^4^ among whom incidence rates of IBD have been increasing significantly.^2^ Further, racial/ethnic minorities might face additional barriers to quality care and positive health outcomes compared with those of nonminority racial/ethnic backgrounds,^7^ including low-income level, challenges due to cognitive capacity or communication abilities, primary language other than English, low education levels, rurality, immigration status, degree of acculturation, and older age (ie, 65+ years old).^8, 9^ The cumulative effects of multiple barriers to quality care can lead to increased vulnerability, higher physiological burden, and deleterious effects on health.^10^ Additionally, while research is limited, current data indicate that LHL is an independent predictor of racial/ethnic disparities in health behaviors (eg, poor medication adherence and more emergency care visits), access to care, self-rated health, and early mortality.^3, 4^

In this issue, Dos Santos Marques et al present data on health literacy among 175 English-speaking adult patients with IBD who were treated at a tertiary-referral IBD center in Alabama (citation to be added once available). The authors’ primary goal was to evaluate health literacy in patients at the IBD center using New Vital Sign (NVS), a validated assessment of health literacy that includes tests of reading and numeracy. Overall, 24% of participants were classified as having LHL based on the NVS. Among participants who identified as African American, 47.5% were found to have LHL compared with 17.0% of their Non-Hispanic White counterparts. Further analyses indicated that both African American race and older age were uniquely associated with LHL, while African American participants not only experienced more LHL overall, the influence of age on health literacy was also more impactful for this group. Thus, LHL was most common in older African American participants.

The authors conclude that increased screening of health literacy early in IBD patient care is necessary to better serve IBD patients with LHL and ultimately improve postsurgical outcomes. In addition to early screening, Dos Santos Marques et al suggest the use of literacy-based interventions for identified patients at various points in their care. The authors’ findings support the need for early assessment of health literacy levels and highlight a key opportunity for healthcare providers to help improve general health or disease-specific knowledge during clinical encounters and tailor IBD services in culturally and literacy-sensitive ways.

As is highlighted in the work by Dos Santos Marques et al, LHL can be intertwined with race and/or ethnicity. When considering factors that contribute to LHL among patients with IBD, healthcare providers and researchers alike would do well to conceptualize LHL as but one factor among a constellation of socioecological challenges that can place individuals at higher risk for poor overall health. Some authors advocate for increased recognition of how historical factors, such as systematic oppression, have impacted many communities’ access to healthcare, education, secure housing, and healthy food sources, which, taken together, can lead to the development or maintenance of LHL.^9, 11^

In the context of clinical encounters, healthcare providers might engage with patients in a way that circumvents historically- or institutionally based power structures in order to support self-care, independence, and foster a trusting partnership. One well-recognized way to disrupt imbalanced power structures in healthcare and develop effective and egalitarian patient–provider relationships is to encourage patients to express concerns and ask questions about their health and treatment plans. Additionally, it is important to consider not only the clinical encounter but also the office setting/environment and any patient materials when providing culturally and literacy-sensitive care. In Table 1, we present examples of ways clinicians can provide LHL-sensitive care and the rationale behind these strategies. These strategies may be particularly important in the context of the ongoing COVID-19 pandemic, where LHL or other socioeconomic differences may be magnified by internet signal, childcare availability, or other new aspects of the clinical encounter.

**Table 1.** Strategies for Improving Care for Patients Who Have Low Health Literacy

LHL Strategy	Rationale
Office Environment	
Choose photos and reading material that are relevant to or represent the patient population, such as material in languages commonly spoken, or that center on local leisure activities.	Patients might feel more like they belong in the office space and that the providers are accustomed to caring for others like them.
If possible, consider having staff who represent the cultural makeup of the local community(ies) from which patients come. Another strategy would be to include clear signs of interpreter services.	Patients might then feel more included, seen, and acknowledged upon arriving, which could encourage them to be less reticent during the clinical encounter.
Clinical Encounter	
Ask about health literacy with questions such as: “Many people find medical terms hard to understand. Do you ever need (or get) help with filling out forms or reading prescription labels?” “When you have to learn something new, how do you prefer to learn it – watching a video, hearing someone explain it, reading, talking to people?”	Helps to normalize the challenges patients might face in reading and interpreting health information and care instructions, as well as to assist healthcare providers to determine the best mode of communication for patient education.
Ask culturally sensitive questions about health decision-making, such as: “Some people have a certain person(s) in their lives who they like to talk to about decisions. Do you have someone like that you’d like to either include in a visit or talk to before you make a decision? Who is that person?”	Assists healthcare providers and patients to determine together who might be helpful to include in their visits and discussions about the patient’s health.
Ask about how the patient’s cultural background, identity, or values might support or impede health management,^15^ such as: “Sometimes, aspects of people’s background or identity make it easier or harder to manage their illness. Is there anything about your background that impacts your illness or your ability to take care of yourself?	Assists healthcare providers and patients to identify potential strengths or protective factors, as well as barriers to care.
Education/Patient Materials	
Design patient materials using simple words, bigger fonts, and bullet points.	Ease of reading for non-native speakers, individuals with lower education levels, or individuals with interfering medical or psychiatric conditions.
Consider integrating culturally relevant stories and metaphors into education materials.	Helps patients relate to the material and feel more comfortable sharing health beliefs.

In addition to the need to improve our clinical interactions with individuals with low LHL, researchers can improve their outreach to and interactions with participants. As Dos Santos Marques et al note in their limitations section, their survey response rate was notably lowest among patients with LHL. This finding is consistent with data indicating that individuals with LHL participate less in research,^12^ even after adjusting for age, gender, race, income, education, and other characteristics. This overlooked impact on research participation is worth investigating further, as it could substantially expand epidemiological understandings of disease, relevant risk factors, and/or important variables in disease management. Current research in clinical trials in oncology^13^ suggests that while study sites with diverse populations are more likely to engage in outreach and present materials in multiple languages, these efforts remain insufficient. Similarly, a study utilizing focus groups to understand research coordinators’ experiences with recruitment of underrepresented groups identified barriers related to translation, literacy, family composition, and severity of medical diagnosis.^14^

## CONCLUSIONS

Overall, Dos Santos Marques et al’s research builds on an important topic in IBD patient care, finding that consistent with the extant literature, LHL was most common in older African American participants. For clinicians and researchers, this work highlights the importance of (a) ensuring that clinical environments are inclusive and supportive of LHL patients and (b) using special recruitment strategies to ensure participation of people with LHL. It is likely that these clinical and research strategies will not only support patients with LHL but also those with other barriers to care.
